# Transcatheter arterial chemoembolization of apatinib and camrelizumab (SHR1210) against liver metastasis from hepatic neuroendocrine tumor: a case report

**DOI:** 10.3389/fonc.2024.1278340

**Published:** 2024-02-07

**Authors:** Ruobing Qi, Wenhua Yang, Sixian Zhu, Jie Mao, Bei Yang, Anhui Xu, Qiang Fu

**Affiliations:** ^1^ Department of Oncology, Tongji Hospital, Tongji Medical College, Huazhong University of Science and Technology, Wuhan, Hubei, China; ^2^ Second School of Clinical Medicine, Tongji Medical College, Huazhong University of Science and Technology, Wuhan, China; ^3^ Department of Radiology, Tongji Hospital, Tongji Medical College, Huazhong University of Science and Technology, Wuhan, Hubei, China

**Keywords:** NET G2, HCC, camrelizumab (SHR1210), apatinib, PD-1, TACE

## Abstract

In this case report, we present the case of a 46-year-old woman with a hepatic neuroendocrine tumor (NET G2)-induced liver metastases. Initially, the left lateral lobectomy of the liver was performed. The post-operative pathological examination revealed NET G2, leading to the post-operative recovery with a general review. Further, the re-examination of liver magnetic resonance imaging (MRI) showed post-operative changes in the tumor of the left lateral lobe, with multiple liver masses and possible metastasis. Thus, the liver interventional therapy and apatinib-based targeted therapy based on the “camrelizumab + apatinib” regimen were performed, respectively. The 20-month follow-up indicated a slightly increased hepatic hilum and retroperitoneal lymph nodes, accompanied by hand-foot syndrome. Eventually, the overall condition continued to relieve, indicating that the combined treatment could substantially improve the NET G2 conditions-associated liver metastasis.

## Introduction

Neuroendocrine tumors (NETs) are rare, heterogeneous, and typically indolent tumors that often originate primarily in the lungs and gastrointestinal tract (GIT). These rare NET are vascularized tumors expressing many pro-angiogenic molecules (1). Over the past three decades, the incidence rates have been on an upward trend (2), accounting for an increase in the rate of occurrence by 5-fold. Notably, the selection of a chemotherapeutic regimen to act against NETs is predominantly based on the prolonging effects concerning the progression-free survival (PFS), time to progression (TTP), overall survival (OS), and objective tumor response rates (ORR). Moreover, these heterogeneous NETs are often prone to liver metastases (NET-LM), in which the patients affected with NETs are typically diagnosed with intermediate or advanced tumors.

Currently, the most effective method for treating NET-LM is transcatheter arterial chemoembolization (TACE) (3). TACE has emerged as one of the specific types of chemoembolization approach used to block the short blood vessels that nourish the liver (hepatic artery) with oxygenated blood for cancer therapy (4). Further, the NET-LM progression is often dependent on T-cell exhaustion, in which the programmed cell death protein 1 (PD-1)-based pathway is a major negative regulator of T-cell survival and proliferation in the tumor microenvironment (TME) (5). Along this line, the combination of blockade of PD-1 and a vascular endothelial growth factor receptor 2 (VEGFR2) inhibitor (apatinib) *in vitro* enhanced the lethal effect of multiple antigen-specific cell therapy (MASCT) (6). Several reported studies indicated that the relationship between angiogenesis and anti-tumor immunity in TME was bidirectional (7–9). In this context, the application of anti-angiogenic drugs could eliminate the immunosuppressive effects of TME. Moreover, these anti-angiogenic drugs combined with immunotherapeutics could improve their performance efficacy (10–12). In an instance, the TACE therapy combined VEGFR inhibitors and PD-1 inhibitors in patients with NET-LM demonstrated better clinical efficacy and manageable safety compared with TACE alone (13). In addition, triple therapy with no obvious toxicity or controllability (13) has a high tumor remission rate and transformation resection rate (14).

Considering these aspects, herein, we report a case of TACE-combined apatinib and camrelizumab (SHR1210) administration to act against liver metastases from hepatic NET G2 tumors.

## Case report

**Figure 1:**
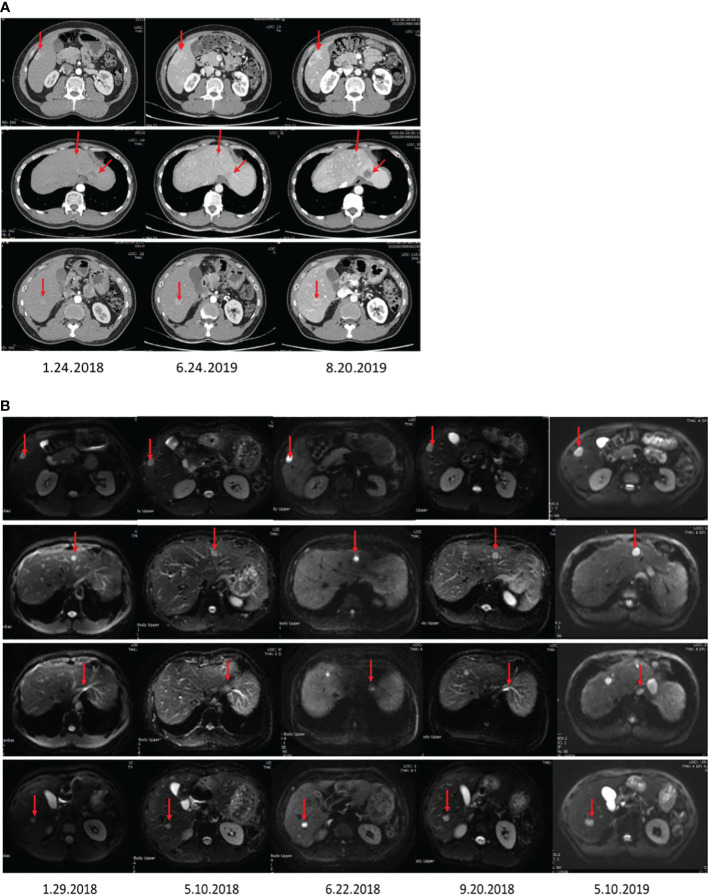
The images display the abdominal CT **(A)** and MRI **(B)** before surgery, showing multiple liver nodules.

A 46-year-old female patient with complaints of slight fullness discomfort in the right upper quadrant of the abdomen was admitted to Tongji Hospital, Tongji Medical College, Huazhong University of Science and Technology (Wuhan, China) for liver nodule on January 24, 2018. During the preliminary physical examination, the patient had no fever and had normal vital signs. We were informed that the patient had no previous medical history, including post-operative drug history, as well as family medical history. Accordingly, computed tomography (CT) and magnetic resonance imaging (MRI) examinations were regularly performed to monitor the health condition (**Supplementary Table S1**), revealing several fast-growing masses in the liver (**Figure 1**). A written informed consent was obtained from the patient prior to publication of the present study.

**Figure 2:**
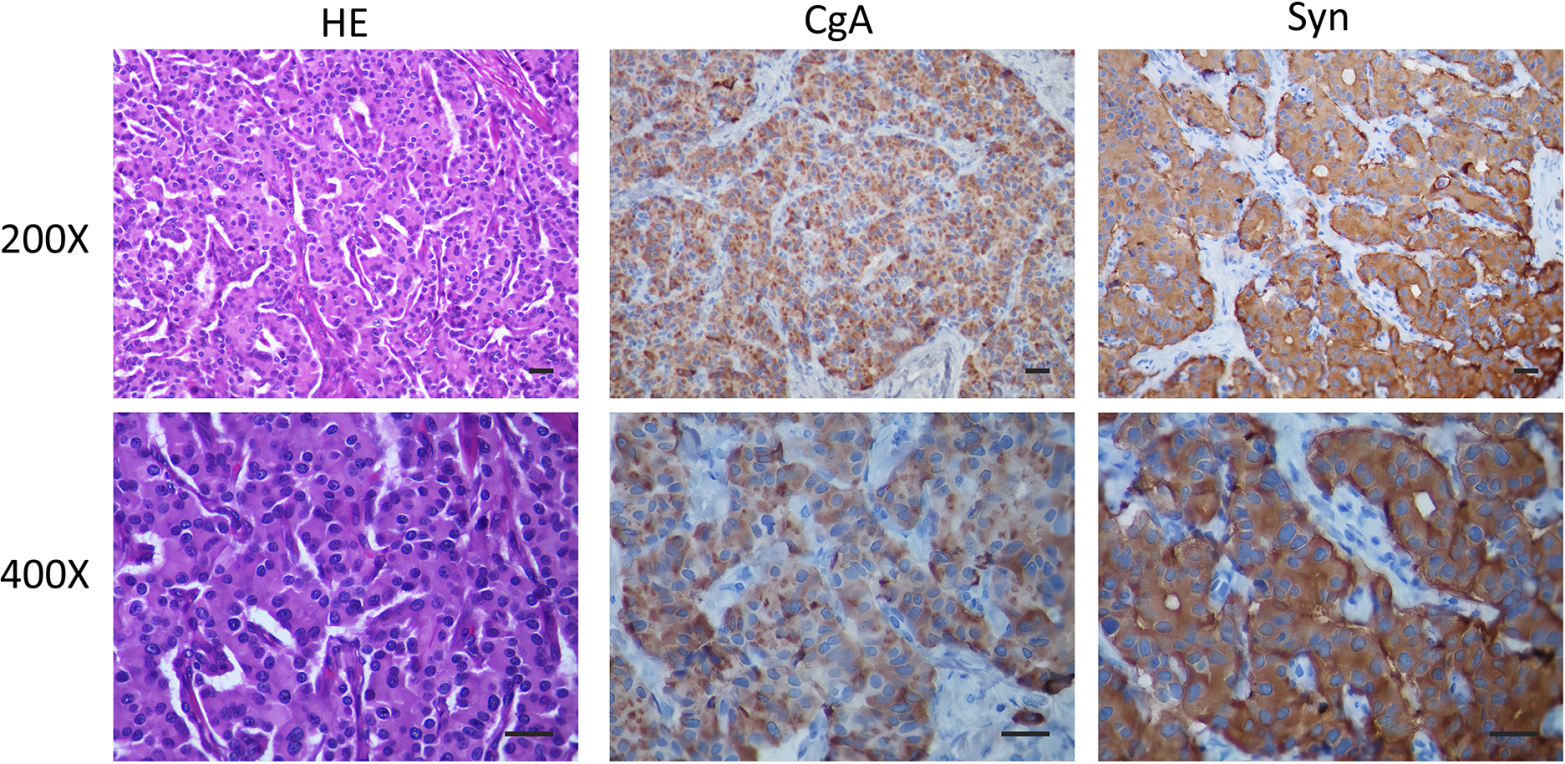
The post-operative liver pathological observations show a liver combined with an immunophenotype consistent with a NET. The imaging techniques include H&E (Conventional HE staining), CgA (Immunohistochemistry CgA), Syn (Immunohistochemistry Syn), Scale bar of 50 μm. The automatic immunohistochemical staining experiment was performed on the Dako Autostainer link 48 (Elabscience Biotechnology Inc. Houston, TX, USA) instrument with appropriate Elabscience ^®^ RTU antibodies.

**Figure 3:**
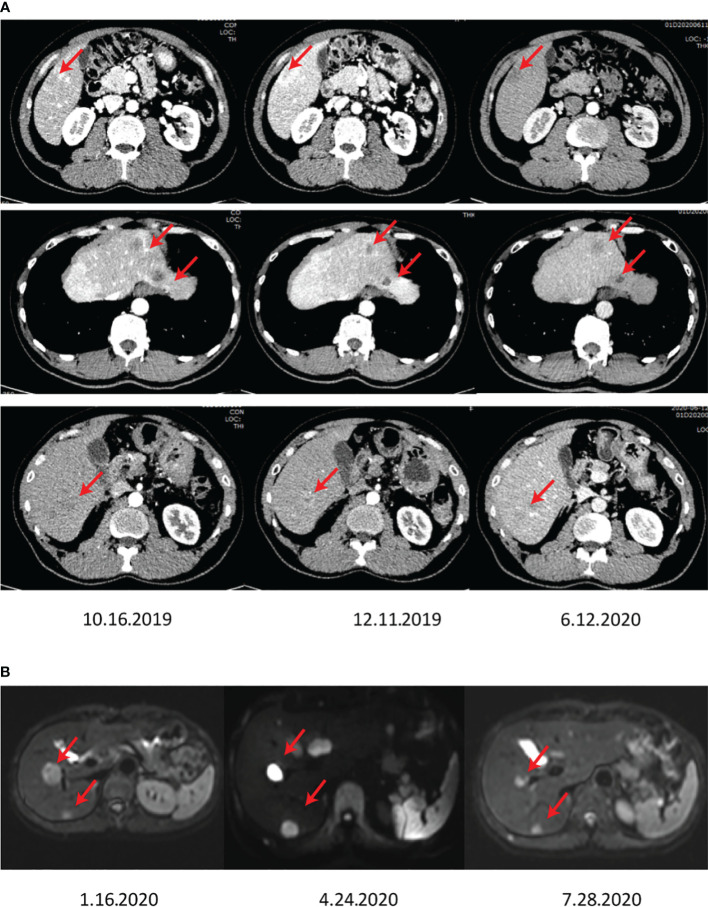
The images show the abdominal CT **(A)** and MRI **(B)** after surgery, showing growing lesions in the residual liver.

**Supplementary Table S2** shows the timeline of the treatments received by the patient. According to the records, the patient had undergone a liver lobectomy on October 13, 2017, in Tongji Hospital (Wuhan, China). Afterward, the patient had no abdominal pain, abdominal distension, nausea, vomiting, or skin yellowing. The post-operative pathological results of the liver displayed that the liver combined with immunophenotype was consistent with neuroendocrine tumor (NET, G2). The patient had a positron emission tomography/X-ray computed tomography (PET-CT) whole-body examination on December 5, 2017, and exhibited no significant lesions in other parts of the body except the liver (**Figure 2**). However, the gastroscopic and enteroscopic examinations identified no primary lesions in the rest of the body, including GIT. Consequently, we speculated that the possible primary foci originated from the liver or hidden parts of the stomach, intestines, and pancreas. Further, a CT follow-up scan was performed on December 11, 2019, in Tongji Hospital, revealing a post-operative alteration and several metastases in the residual liver (**Figure 3A**).

Considering NETs-based metastases, the patient underwent primary TACE (Loplatin) 2 months after admission. It should be noted that the reagent was stored at 2-8°C, dissolved with 5 ml of water for injection before use, and should be used within 4 hours. According to the body surface area, the reagent (50 mg/m^2^) was intravenously injected once, and complete recovery of hematotoxicity or other clinical side effects was monitored, usually at an interval of 3 weeks. In the case of the slow recovery from side effects, the dosing interval must be adjusted longer. After the treatment of the first TACE, the patient showed slight bleeding symptoms. Considering the multiple residual tumors, a second TACE was performed on April 29, 2020, in Tongji Hospital. Further, mild PES conditions post-operation were observed, including mild abdominal distension, no significant vomiting, right upper abdominal pain, and no significant fever. Owing to the advancement of the disease and the multiple nodules of the patient, apatinib at a dose of 250 mg was administered once a day, commencing after the first TACE. However, a rapidly progressing 1.7 x 0.9 cm^2^ mass with a high DWI signal in the subcapsular posterior inferior segment of the right hepatic lobe was observed by MRI screening on July 28, 2020, in Tongji Hospital (**Figure 3B**).

**Figure 4:**
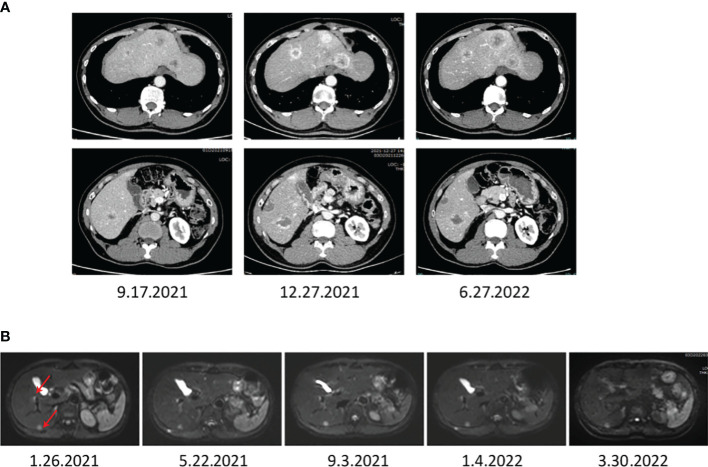
The images display the abdominal CT **(A)** and MRI **(B)** after a combination treatment of TACE, anti-PD-1 antibodies, and apatinib, showing that the liver metastasis of NETs diminished significantly.

The recurrent NETs were suspected, and an anti-PD1 therapy (camrelizumab 200 mg intravenous once every 3 weeks) was prescribed to the regimen on July 31, 2020, in Tongji Hospital. Typically, camrelizumab has been shown to offer high efficacy and acceptable toxicity in pre-treated Chinese patients with advanced liver cancer. Moreover, an MRI investigation after approximately three months of starting combination treatment showed that the intrahepatic tumors were significantly diminished. Further, the regular CT follow-up scans revealed the diminished hepatic tumor sizes in the residual liver, confirming a critical pharmacodynamic response to the combination therapy (**Figure 4**). Nevertheless, a new 2.1 x 1.5 cm^2^ mass was detected in the spleen by MRI monitoring on December 3 in 2021 (**Supplementary Figure S1**) in Tongji Hospital.

Further, the patient was last examined on March 20, 2022, after 2 years since the start of combination therapy and 5 years after diagnosis. As anticipated, the patient was in good health condition, and MRI follow-up monitoring validated a sustained response (**Supplementary Figure S2**, from 2020 to 2022, the T1 and T2 time phases of MRI, marked by red arrows, a significantly reduced liver lesion). These follow-up investigations showed multiple nodules in the liver and nodules in the spleen, which were considered tumors and post-treatment changes, roughly similar to the previous MRI. The hilar and retroperitoneal lymph nodes were slightly increased. Moreover, the laboratory tests showed serum AFP levels of 3.5 mg/l, total bilirubin of 26 mmol/l, albumin of 44 g/l, and prothrombin time (PT) of 14.2 sec. Notably, no evidence of hepatic encephalopathy or ascites was observed, and the patient’s Child-Pugh score was 5. The major toxicity of treatment was found to be hand-foot syndrome. Nevertheless, no other adverse events occurred during the following 2-year treatment period. Overall, the health condition of the patient was quite stable and much improved compared with the previous physical conditions. After March 2022, the patient continued with the intermittent oral apatinib treatment due to its stable effect during the previous treatment procedure, which was discontinued in June 2022. Nevertheless, in April 2023, the local hospital review suggested the progression of liver lesions. Then, she approached our hospital for further treatment and was another time given the TACE + apatinib + camrelizumab. InSupplementaryMay 2023, the physical examination results suggested a significant reduction of intrahepatic lesions. Nonetheless, the patient showed no enduring hand-foot syndrome, and the patient was in remission mode by the latest review with no significant complications.

## Discussion

Characteristically, the NET-LM progression is dependent on T-cell exhaustion. Along this line, the PD-1 pathway is a major negative regulator of T-cell survival and proliferation in the TME (5). Considering the mechanism, the blockade of PD-1 and a VEGFR2 inhibitor (apatinib) *in vitro* could significantly enhance the lethal effect of MASCT (6). Based on the understanding of the biology of NETs and critical cues in the TME, the most commonly used treatment methods include surgical interventions (including endoscopic surgery), interventional radiotherapy (mainly for liver metastases), peptide receptor-mediated radionuclide therapy (peptide radioreceptor therapy, PRRT), small molecular drugs-based chemotherapy, biological therapy, and molecular targeted therapy. In this framework, the commonly used therapeutic drugs include somatostatin analogs (octreotide and lanreotide), interferons, targeted drugs (everolimus or sunitinib), and cytotoxic chemotherapeutics, among others. In the present case report, the patient was subjected to a PET-CT whole-body examination on December 5, 2017, indicating no lesions in other parts of the body except the liver. Further, a whole-body bone imaging was performed on May 24, 2021, displaying that the whole-body bone structure was clearly visible in blue, with no obvious abnormal radioactive distribution. In addition, no signs of tumor bone metastasis were observed. A CT scan of the parathyroid gland, lung, brain, and GIT, as well as an ultrasound of gynecology, pancreas, and thyroid gland, were performed, showing no tumor lesions. Thus, we reasonably assumed that the NET in the body of the patient was of hepatic origin. Nevertheless, the patient refused to do PET-CT again in the later follow-up due to financial reasons. Thus, we had no records of PET-CT and growth inhibitor receptor phenomena at the site of origin. In conclusion, we decided to treat the patient locally in the liver accordingly. Nevertheless, the patient refused to use the aforementioned drugs for financial reasons, as these expensive treatment procedures could not be reimbursed by medical insurance organizations in China. In contrast, TACE therapy has emerged as one of the commonly used strategies in China for the treatment of primary liver cancer or liver metastases, which can be reimbursed by medical insurance. Considering the capability of TACE therapy for liver cancer and liver metastases, the combination of TACE and PD-1 and VEGFR inhibitors would offer better and more manageable clinical efficacy for the treatment of hepatic neuroendocrine tumors (NET G2) compared with TACE alone. Moreover, these treatment options could offer exceptional safety and no significant toxic effects. Considering these attributes, we adopted the combination of TACE, apatinib, and anti-PD-1 antibody in the treatment of NET G2 liver metastases, showing an exceptional anti-tumor effect.

According to the Barcelona Clinic Liver Cancer (BCLC) staging system, TACE is often preferred to treat intermediate-stage liver cancer, including unresectable nodular liver cancer without extrahepatic spread. This innovative TACE-based therapy is a special type of chemoembolization used to block the short blood vessels that supply the liver (hepatic artery) with oxygenated blood while treating cancer (15, 16). TACE can be performed using two techniques: conventional TACE (cTACE) and TACE-based drug-eluting beads (DEB-TACE) (17). By delivering various small-molecular chemotherapeutic drugs and embolic agents to the blood supply arteries of hepatocellular carcinoma, it controls the tumor growth by blocking the blood supply to the tumor site and causing ischemia and hypoxia (4). Nevertheless, half of the liver cancer patients must choose systemic therapy, as the TACE utilizes the dynamics of liver blood flow, improving the survival rate of patients (4). Moreover, VEGF and FGF expressions upregulated by TACE can be effectively inhibited by tyrosine kinase inhibitors (TKIs), leading to better clinical outcomes in combination with TKIs (18, 19). In this framework, several reports indicated that the combination therapy of ICIs and anti-angiogenic drugs presented better anti-tumor activity and significantly improved survival rate compared with monotherapy (13).

In addition, we believe that the principle of drug selection for cancer therapy is mainly to prolong various attributes of patients, such as PFS, TTP, OS, and ORR. In this regard, several reports applied the VEGFR inhibitor, *i.e.*, surufatinib, for treating NETs, biliary tumors, and gastric cancer, among others (20–22). Due to its comprehensive coverage of VEGFR1/2/3, fibroblast growth factor receptor 1 (FGFR1), and colony-stimulating factor-1 receptor (CSF-1R), strong biological activity, good kinase selectivity, and suitable pharmacokinetic properties, among others, surufatinib offered anti-angiogenesis, improved tumor immunosuppression and synergistic effect of combined immunotherapeutic effects. Owing to these effects, the experimental results indicated that 84% of patients achieved tumor regression. In comparison, 36% of patients attained tumor regression, in which more than 10% of the SANET-ep reached the primary efficacy endpoint and terminated early. Some attributes included mPFS of as high as 9.2 months, ORR of as high as 19%, and rapid onset of action (20). Notably, drugs suitable for tumor treatment can be screened through the numerical comparison of these important indicators. Moreover, surufatinib combined with PD-1 with additional surgical resection could offer a favorable outcome for the control of multiple solid tumors, with good safety and no hand-foot skin reaction. Along this line, our findings were similar to the results of the reported studies using alternative drugs.

In this case report, we replaced surufatinib with apatinib, which resulted in similar effects, using a combination of TACE, apatinib, and anti-PD-1 antibody, leading to a favorable control over tumor growth. Initially, our patient was treated with TACE to explore the phenomenon by treating a small cyst in the left kidney. Further, the patient was given a combination of apatinib and anti-PD-1 antibody. The combined treatment showed improved therapeutic outcomes in the patient. These findings were consistent with the previous studies (23–26), showing a beneficial additive effect of apatinib on TACE treatment. In another instance, the addition of anti-PD-1 antibody therapy to apatinib increased the potent anti-tumor activity (1). During treatment, the data, including early detection of disease, monitoring of tumor heterogeneity, identification of therapeutic targets, real-time treatment response, early detection of tumor recurrence, and dynamic assessment of resistance development helped us in monitoring the patient’s progress in our case study to ensure the treatment appropriateness (27). Typically, apatinib, as a new oral small molecule TKI, can effectively inhibit VEGFR and FGFR, among others, in cancer patients. The unique combination of molecular activities includes the inhibition of tumor angiogenesis, modulation of tumor immune evasion, and reduction of tumor drug resistance (1). Along this line, the combination of SHR1210 and apatinib in the treatment of advanced NET-LM showed manageable toxicity of two drugs at the recommended single dose (28). In this vein, the reasonable dosage of anti-angiogenic drugs, such as apatinib, could modulate and optimize the TME, contributing to the resistance to anti-PD-1/PD-L1 therapy and enhancing PD-1/PD-L1-based anti-tumor effect In addition, it could significantly delay tumor growth, reducing the number of metastases, and prolonging survival to help treat NET-LM (29). Compared with TACE alone, anti-angiogenic therapy and targeted therapy combined with TACE could reduce tumor volume and vascular density (30), as well as enhance the anti-tumor effect.

## Limitations

Although our case report is based on only a patient, we believe that apatinib and camrelizumab (SHR1210) combined with TACE resulted in effective tumor control and improved the overall health of this patient with NET G2-based liver metastases. In addition, there is a scope in employing some other combined treatment options, including the combination of TACE combination therapy (TACE + radiofrequency ablation, TACE + radiotherapy, TACE + systemic therapy) (31–44) and immune combination therapy (two ICIs, ICIs, and angiogenesis inhibitors, ICIs and local therapy, ICIs and chemotherapy) (45–49).

## Data availability statement

The original contributions presented in the study are included in the article/**Supplementary Material**. Further inquiries can be directed to the corresponding authors.

## Ethics statement

The study was approved by the Tongji Medical College. All procedures performed in studies involving human participants were in accordance with the ethical standards of the institutional and/or national research committee and with the 1964 Helsinki Declaration and its later amendments or comparable ethical standards. Written informed consent was obtained from each patient included in the study.

## Author contributions

RQ: Conceptualization, Data curation, Investigation, Writing – original draft. WY: Conceptualization, Methodology, Writing – original draft. SZ: Conceptualization, Investigation, Methodology, Writing – original draft. JM: Investigation, Methodology, Writing – original draft. BY: Methodology, Writing – original draft. AX: Conceptualization, Investigation, Methodology, Writing – original draft. QF: Conceptualization, Data curation, Writing – original draft.

## Supplementary material

Supplementary Figure 1 The representative MRI images show the liver tumor (marked by the arrow) shrank after a combination treatment of TACE, anti-PD-1 antibodies, and apatinib. **(A–C)** T1WI, **(D–F)** T2WI, **(G–I)** T1WI, and **(J–L)** T2WI.

Supplementary Figure 2 MRI images during the last follow-up show multiple intrahepatic nodules and splenic nodules, similar to those in the previous film, considering the tumor and changes after treatment. Notably, the hilar and retroperitoneal lymph nodes are increased slightly. The patient was in good health, and MRIs confirmed a sustained response at the follow-up visits, such as ~approximately 2-year follow-up visits after the start of the combination therapy and 5 years after the following diagnosis.
