# Supplementary figures and images for: Transcatheter arterial chemoembolization of apatinib and camrelizumab (SHR1210) against liver metastasis from hepatic neuroendocrine tumor: a case report
Source: Front Oncol. 2024 Feb 7;14:1278340. doi: 10.3389/fonc.2024.1278340 (PMC10880017; doi:10.3389/fonc.2024.1278340)

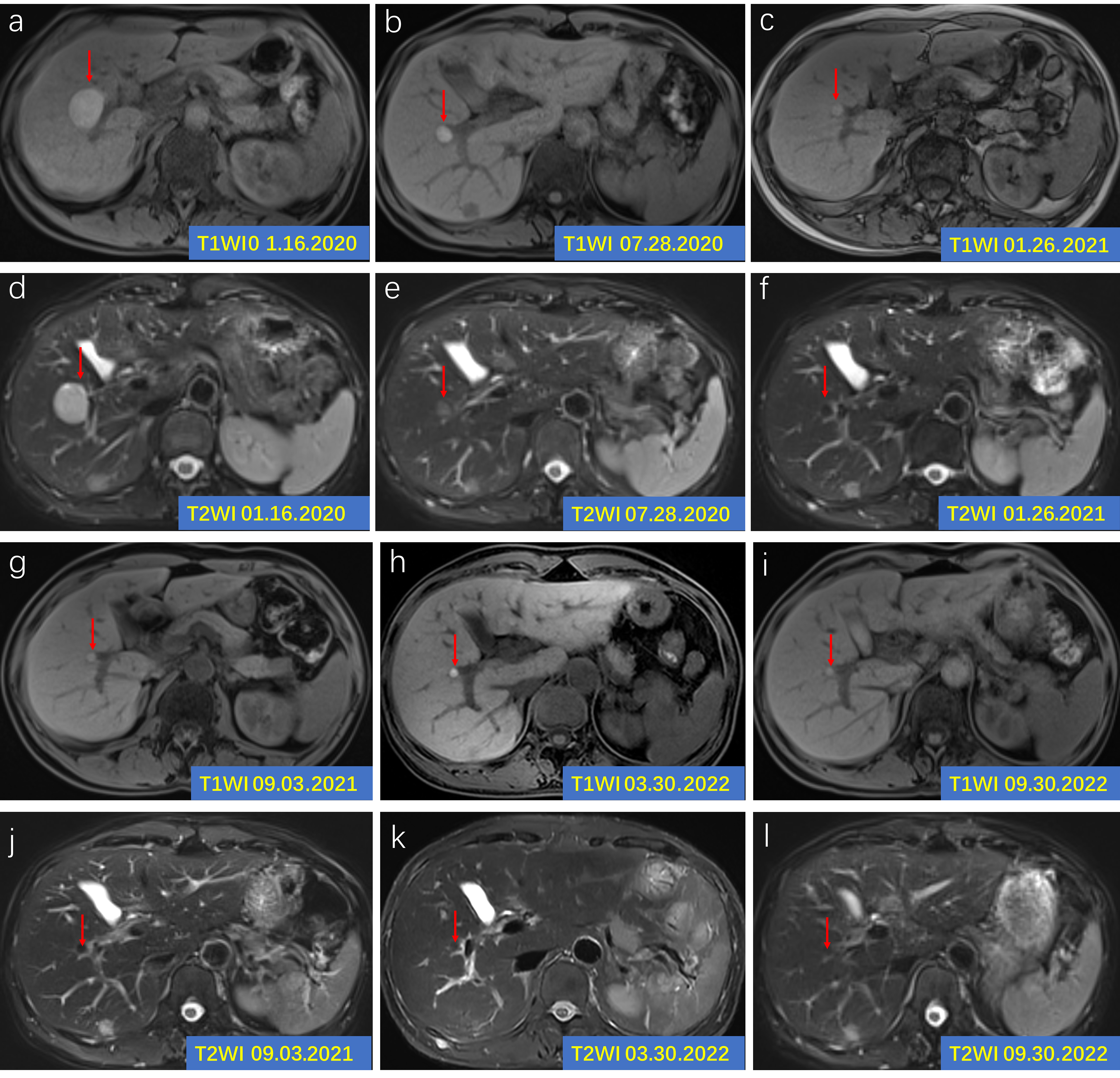

Supplement: Supplementary Figure 1 — The representative MRI images show the liver tumor (marked by the arrow) shrank after a combination treatment of TACE, anti-PD-1 antibodies, and apatinib. (A–C) T1WI, (D–F) T2WI, (G–I) T1WI, and (J–L) T2WI. [file Image_1.tif]

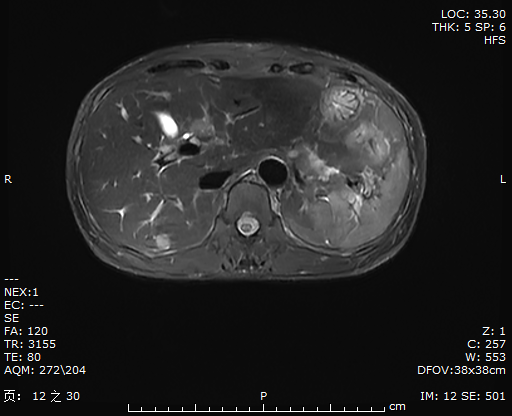

Supplement: Supplementary Figure 2 — MRI images during the last follow-up show multiple intrahepatic nodules and splenic nodules, similar to those in the previous film, considering the tumor and changes after treatment. Notably, the hilar and retroperitoneal lymph nodes are increased slightly. The patient was in good health, and MRIs confirmed a sustained response at the follow-up visits, such as ~approximately 2-year follow-up visits after the start of the combination therapy and 5 years after the following diagnosis. [file Image_2.tif]
