# Supplementary material for: Transcatheter arterial chemoembolization of apatinib and camrelizumab (SHR1210) against liver metastasis from hepatic neuroendocrine tumor: a case report
Source: Front Oncol. 2024 Feb 7;14:1278340. doi: 10.3389/fonc.2024.1278340 (PMC10880017; doi:10.3389/fonc.2024.1278340)
Supplement: Supplementary file 3 [file Table_1.docx]

Table 1. The parameters of MRI performed on 1.5 T (Aera, Siemens Healthcare; Signa HDxt, GE Healthcare) or 3 T (Skyra, Siemens Health care; uMR 780 and uMR 790, UIH; Discovery MR750, GE Healthcare, ).

| Manufacturer | Model | Field Strength | Image sequence | TR(ms) | TE(ms) | FA(D*) | ST(mm) | NEX | SS |
| --- | --- | --- | --- | --- | --- | --- | --- | --- | --- |
| SIEMENS | Aera | 1.5T | T1WI | 6.51 | 2.39 | 10 | 3~3.6 | 1 | GR |
|  |  |  | T2WI | 3550~4735.28 | 83~86 | 160 | 5~6 | 1 | SE |
|  |  |  | DWI b=1000 | 5500~7600 | 58~66 | 90 | 5 | 1 | EP |
|  | Skyra | 3.0T | T1WI | 5.79 | 2.46 | 12 | 3.5 | 1 | GR |
|  |  |  | T2WI | 3560 | 77 | 107 | 4 | 1 | SE |
|  |  |  | DWI b=1000 | 10500 | 52 | 90 | 4 | 1 | EP |
| UIH | uMR 780 | 3.0T | T1WI | 3.57 | 1.61 | 10 | 3.33 | 1 | GR |
|  |  |  | T2WI | 3398 | 78.9 | 120 | 6 | 1 | SE |
|  |  |  | DWI b=0,600,1000 | 3333.0 | 69.0 | 90 | 6 | 1~4 | EP |
|  | uMR 790 | 3.0T | T1WI | 4.08 | 1.49 | 15 | 3 | 1 | GR |
|  |  |  | T2WI | 3575 | 80 | 120 | 5 | 1 | SE |
|  |  |  | DWI b=50~1000 | 3000 | 61.2 | 90 | 5 | 1~8 | EP |
| GE | Signa HDxt | 1.5T | T1WI | 3.49~6.08 | 1.67~3.13 | 12~15 | 5~5.4 | 0.70~0.76 | GR |
|  |  |  | T2WI | 6666.67~9473.68 | 64.88~117.97 | 90 | 6 | 2~3 | SE |
|  |  |  | DWI b=600,1000 | 5152~10000 | 67.90~77 | 90 | 6 | 1 | EP, SE |
|  | Discovery MR750 | 3.0T | T1WI | 3.79 | 1.71 | 12 | 5 | 0.7 | GR |
|  |  |  | T2WI | 10000 | 52.83 | 110 | 6 | 1.5 | SE |
|  |  |  | DWI b=600,1000 | 5454.55 | 52.1 | 90 | 6 | 1 | EP, SE |
