# Supplementary material for: Transcatheter arterial chemoembolization of apatinib and camrelizumab (SHR1210) against liver metastasis from hepatic neuroendocrine tumor: a case report
Source: Front Oncol. 2024 Feb 7;14:1278340. doi: 10.3389/fonc.2024.1278340 (PMC10880017; doi:10.3389/fonc.2024.1278340)
Supplement: Supplementary file 4 [file Table_2.docx]

| **Table 2. Timeline of the treatments that the patient received** | | |
| --- | --- | --- |
| **Time** | **Therapy** | **Results** |
| Oct-2017 | Left liver lobectomy | Pathological examination revealed hepatic neuroendocrine tumor and the patient's clinical condition improved significantly |
| Dec-2019 | - | MRI showed several metastasis in the residual liver |
| Dec-2019 to Apr-2020 | TACE+Apatinib targeted therapy | MRI in Jul-2020 showed A 1.7x0.9cm mass rapidly progressing in the posterior lower segment of the right hepatic lobe under the capsule, with high DWI signal, which were suspected recurrent NETs. |
| Jul-2020 to Mar 2022 | anti-PD1 therapy (camrelizumab 200mg intravenous once every 3 weeks) +Apatinib targeted therapy | The patient recovered well and MRI showed that intrahepatic tumors were significantly reduced |
